# Violence against female sex workers in Karnataka state, south India: impact on health, and reductions in violence following an intervention program

**DOI:** 10.1186/1471-2458-10-476

**Published:** 2010-08-11

**Authors:** Tara SH Beattie, Parinita Bhattacharjee, BM Ramesh, Vandana Gurnani, John Anthony, Shajy Isac, HL Mohan, Aparajita Ramakrishnan, Tisha Wheeler, Janet Bradley, James F Blanchard, Stephen Moses

**Affiliations:** 1 Department of Public Health and Policy, London School of Hygiene and Tropical Medicine, Keppel Street, London, WC1E 7HT, UK; 2 Karnataka Health Promotion Trust, IT/BT Park, 5th Floor, #1-4/, Rajajinagar Industrial Area, Behind KSSIDC Administrative Office, Rajajinagar, Bangalore 560 044, India; 3 Department of Community Health Sciences, University of Manitoba, 750 Bannatyne Avenue, Winnipeg, R3E 0W3, Canada; 4 India Health Action Trust, # 4/13-1, Pisces Building, Crescent Road, High Grounds, Bangalore 560001, India; 5 India AIDS Initiative (Avahan), Bill & Melinda Gates Foundation, Sanskrit Bhavan, A-10 Qutb Institutional Area, Aruna Asaf Ali Marg, New Delhi 110067, India; 6 Unité de recherche en santé des populations, Centre hospitalier affilié universitaire de Québec, Hôpital du Saint-Sacrement, Québec, Canada; 7 Department of Medical Microbiology, University of Manitoba, 745 Bannatyne Avenue, Winnipeg, R3E 0J9, Canada

## Abstract

**Background:**

Violence against female sex workers (FSWs) can impede HIV prevention efforts and contravenes their human rights. We developed a multi-layered violence intervention targeting policy makers, secondary stakeholders (police, lawyers, media), and primary stakeholders (FSWs), as part of wider HIV prevention programming involving >60,000 FSWs in Karnataka state. This study examined if violence against FSWs is associated with reduced condom use and increased STI/HIV risk, and if addressing violence against FSWs within a large-scale HIV prevention program can reduce levels of violence against them.

**Methods:**

FSWs were randomly selected to participate in polling booth surveys (PBS 2006-2008; short behavioural questionnaires administered anonymously) and integrated behavioural-biological assessments (IBBAs 2005-2009; administered face-to-face).

**Results:**

3,852 FSWs participated in the IBBAs and 7,638 FSWs participated in the PBS. Overall, 11.0% of FSWs in the IBBAs and 26.4% of FSWs in the PBS reported being beaten or raped in the past year. FSWs who reported violence in the past year were significantly less likely to report condom use with clients (zero unprotected sex acts in previous month, 55.4% vs. 75.5%, adjusted odds ratio (AOR) 0.4, 95% confidence interval (CI) 0.3 to 0.5, p < 0.001); to have accessed the HIV intervention program (ever contacted by peer educator, 84.9% vs. 89.6%, AOR 0.7, 95% CI 0.4 to 1.0, p = 0.04); or to have ever visited the project sexual health clinic (59.0% vs. 68.1%, AOR 0.7, 95% CI 0.6 to 1.0, p = 0.02); and were significantly more likely to be infected with gonorrhea (5.0% vs. 2.6%, AOR 1.9, 95% CI 1.1 to 3.3, p = 0.02). By the follow-up surveys, significant reductions were seen in the proportions of FSWs reporting violence compared with baseline (IBBA 13.0% vs. 9.0%, AOR 0.7, 95% CI 0.5 to 0.9 p = 0.01; PBS 27.3% vs. 18.9%, crude OR 0.5, 95% CI 0.4 to 0.5, p < 0.001).

**Conclusions:**

This program demonstrates that a structural approach to addressing violence can be effectively delivered at scale. Addressing violence against FSWs is important for the success of HIV prevention programs, and for protecting their basic human rights.

## Background

The advent of the human immunodeficiency virus (HIV) pandemic has led to increased awareness about the violence experienced by women [1,2] and by female sex workers (FSWs) worldwide [3,4]. Research on domestic violence from developing countries suggests that anywhere from 10-60% of married women of reproductive age report experiencing some form of domestic violence [2], and that domestic violence is strongly associated with physical and mental health morbidity, including homicide, suicide, physical injuries and emotional distress [5,6], as well as HIV seropositivity [1,7,8].

FSWs are frequently marginalised from society due to sex work lacking social or moral approval. In addition to experiencing physical and sexual violence from their intimate partners [9], they can also experience violence from others in their personal and working lives, including clients, pimps, madams and the police [4,10,11]. Compared with the general population, HIV/STI prevalence rates are frequently higher among FSW populations and their clients due to high partner turnover and concurrent sexual partnerships, leading to substantial HIV/STI transmission, particularly when condom use is low [12]. As well as negatively impacting on their mental health and emotional wellbeing [11,13], violence against sex workers can heighten their vulnerability to HIV and other sexually transmitted infections (STIs) through multiple mechanisms: (i) coerced sex is rarely protected; (ii) coerced sex can result in injuries that can increase the transmission of STIs, which in turn can increase the risk of HIV transmission [14]; (iii) men who are sexually violent may be more likely to have multiple partners and be infected with HIV and/or STIs [8,15,16]; (iv) sex work is usually illegal, and thus reporting of sexual and physical violence to the authorities is difficult [3,11], meaning violence can continue unchecked; (v) the fear of violence from regular partners (husbands/lovers) resulting from inadvertent disclosure of sex work can deter sex workers from negotiating condom use with these partners and from accessing sexual health services [13,17]; and (vi) mental health morbidity arising from violence can reduce the ability of sex workers to negotiate condom use and to access STI services for testing and treatment.

India is experiencing an emerging, predominantly heterosexual HIV epidemic, with approximately 2.5 million individuals infected [18,19]. Although sex work *per se *is not illegal in India (the emphasis of the Indian Immoral Traffic Prevention Act, 1956 being on penalising the act of solicitation, as well as penalising those who profit from sex workers such as madams, pimps, brothel keepers and traffickers, rather than penalising sex work itself), the majority of police officials and sex workers wrongly understand sex work to be against the law [20]. In a setting where gender-based domestic violence is common [8,21,22], marital rape could not (until 2008) be prosecuted, and sex work is widely perceived as illegal and immoral, sex workers often feel that they have little recourse against violence and rape, and the perpetrators of violence against sex workers are unlikely to be punished for their actions [23].

Karnataka state in south India has a population of approximately 54 million and ranks among the top four states in India with regard to HIV epidemic severity [24]. Although the estimated HIV/AIDS prevalence among the general population is approximately 1%, rates among FSWs have reached >25% in some districts. The Karnataka Health Promotion Trust (KHPT), supported by the Bill & Melinda Gates Foundation, has worked since 2003 to deliver targeted HIV preventive intervention programs to over 60,000 FSWs and 20,000 MSM (men who have sex with men)/transgenders in 21 of the 30 districts in the state. FSWs in Karnataka state are often from the lower castes, and are often poor, uneducated, and may have children to support, leaving few economic alternatives for survival if they are deserted by their husbands or become widowed [25]. In our early dialogue with sex workers, it became clear that violence was a key concern. In addition, finding shelter, food and child-care concerns were major priorities, whereas health issues rarely featured. The program realised that physical and sexual violence against the sex worker community could potentially undermine the efforts of HIV prevention programming in multiple ways. The violence experienced by sex workers was considered a priority issue to address, as part of the provision of sexual health education and services. The program therefore created a framework and program to do so, beginning in 2005. In this paper we provide details of a multi-layered strategy involving policy makers, secondary and primary stakeholders, to stem and address violence against the sex worker community as part of a wider HIV intervention program. We then examine the impact of these violence intervention efforts on levels of violence against FSWs, and examine associations between violence and condom use, HIV/STI rates and exposure to the HIV prevention program components.

## Methods

### Violence Interventions

#### (i) Policy level advocacy

At the beginning of the project, meetings were held with senior government officials to advocate for HIV/AIDS as a key social and economic issue. As a result, HIV/AIDS coordination committees were established in each district, comprising the heads of all government development departments and the police department, as well as representatives of the sex worker population and PLWHAs (people living with HIV/AIDS). The committees then worked in partnership with the HIV prevention program to embed HIV prevention and care policies throughout government department activities. They also provided an important platform through which sex workers could directly raise concerns such as violence and harassment with government and police officials, and this in turn helped to generate empathy among government and police officials towards the sex worker community.

#### (ii) Secondary stakeholders: police, lawyers and the media

To address police violence, the program worked in partnership with district police heads and sex workers to deliver state-wide training to over 12,000 police officers, representing over half of all police personnel in the project districts. This training aimed to raise awareness of HIV/AIDS, generate empathy among police officials towards sex workers, and provide clarity regarding the law and sex work. In addition, the HIV prevention program brought together a group of human rights lawyers who volunteered to work with the sex worker community to provide legal literacy, and bring perpetrators of violence to justice. To address the bigger issue of social stigma, state-wide HIV/AIDS awareness and sex worker sensitisation training was provided to over 2000 journalists, who reported in both English and the local language, *Kannada*. This aimed to raise awareness and generate empathy among the general population around issues facing sex workers.

#### (iii) Primary stakeholders (sex workers)

A key component of the HIV prevention program has been to identify sex workers through peer education outreach, and to bring the sex worker community together through a process of community mobilisation, which allows them to work together as a group to address the problems that they face [26]. At the onset, the program opened drop-in centres in each of the project districts, where sex workers can come to rest safely, meet other sex workers, and take part in community activities, including literacy and other classes [27]. This interaction was geared to contribute to an increased sense of self-worth and raise self-esteem for individuals. Community mobilisation activities focused on addressing feelings of powerlessness and isolation, providing fora for practical actions, and advocating against discrimination, stigma, wrongful arrest, violence and harassment.

To provide legal literacy and educate FSWs about their legal rights, the program worked in partnership with human rights lawyers to deliver legal empowerment workshops to over 25,000 FSWs across the state. In order to support sex workers who had been subjected to a violent attack, 24-hour crisis management teams were also established in each district, comprising peer educators, lawyers and NGO project staff. Sex workers were provided with an emergency contact telephone number of a designated team member to call in the event of violent attack, wrongful arrest or sexual assault. This system provided an immediate response in times of crisis, and the teams advocated on the victim's behalf to pursue justice against perpetrators of violence.

### Integrated behavioural and biological assessments (IBBAs)

To evaluate the impact of the HIV prevention program, integrated behavioural-biological assessments (IBBAs) were conducted at baseline and follow-up on random samples of FSWs in four selected districts in Karnataka (Belgaum, Bellary, Shimoga and Bangalore Urban). Resources were only sufficient to conduct IBBAs in this number of districts, so they were selected purposively, based on Karnataka's socio-cultural regions and the size of the high-risk populations. HIV prevention programs were initiated in these four districts between April 2004 and October 2005, with baseline IBBAs conducted 12-16 months after program initiation, and follow-up surveys completed 33-37 months later [28].

Sample size calculations were designed to detect a 10-15% increase in condom use with 90% power and alpha error of 5%, assuming a baseline value for consistent condom use with commercial clients of 50%. Thus it was estimated that 385 participants (rounded to 400) were required for each cross-sectional survey round in each district. In Bangalore urban, two sampling domains were utilized (street-based FSWs and non-street-based FSWs), with a target sample of 400 FSWs in each domain for each round. Following baseline mapping estimates of FSWs in each district, a probability sampling method was used with two different sampling methods adopted: (i) conventional cluster sampling was used for FSWs selling sex at home, or in brothels, lodges and dhabas, where the population of FSWs was relatively stable; and (ii) conventional time-location cluster (TLC) sampling was used for street-based FSWs. The same sampling technique was used for baseline and follow-up surveys. Face-to-face interviews were conducted by trained field work assistants in the local language, *Kannada*, using a comprehensive behavioural questionnaire [27]. As well as questions about demographic characteristics, sex work, sexual behaviour, condom practices, and use of the project drop-in centre and sexual health clinic, the questionnaire contained the following question about violence: *"In the past one year, were you ever beaten or otherwise physically forced to have sexual intercourse with someone even though you didn't want to?"*. Blood and urine samples were also taken from participants to test for HIV, syphilis, gonorrhea and chlamydial infection, as previously described [27].

### Polling Booth Surveys (PBS)

Polling booth surveys were designed to minimise reporting bias (compared with methods such as IBBAs which use face-to face interviews) [29-31], and contained a subset of 23 questions derived from the IBBA questionnaire, including the question on violence. Each year, 12-21 PBS were conducted in each of 13 districts, with 10-15 FSWs participating in each PBS. These 13 districts represented all of those that were taken up originally by the program, and because relatively few resources were required to conduct PBS, more districts and FSWs could be included. The number of surveys conducted in each city/town was calculated proportionate to the number of FSWs contacted by the project in the previous six months, and 20 FSWs per PBS were randomly selected from lists provided by the projects of all FSWs contacted. The project's computerized management information system maintained a list of all FSWs contacted, either through peer outreach and/or clinic visits, along with the date of contact. This list formed the sampling frame for the selection of FSWs for the PBS. Although demographic data were not collected in the PBS, the women sampled are unlikely to have differed substantially from those who participated in the IBBA.

PBS were conducted by trained field work assistants in the local language, *Kannada*. Participants were separated from the interviewer and each other in a "polling booth" environment, and provided with green ("yes"), red ("no") and white ("not applicable") boxes, along with a pack of numbered cards (matching the question number) stacked in serial order. As each question was read out, participants either posted the matching card in the coloured box appropriate to their answer, or kept the card to one side if they did not wish to answer the question. All answers were unlinked and anonymous.

### Ethical Considerations

Participation in both the PBS and IBBAs was by informed consent. Statutory approval for the conduct of the IBBAs and their protocols was obtained from the Government of India's Health Ministry Screening Committee (HMSC). All studies were approved by the Institutional Ethical Review Board of St. John's Medical College, Bangalore, India, and the Health Research Ethics Board of the University of Manitoba, Winnipeg, Canada.

### Statistical analyses

All statistical analyses were performed using survey data analysis techniques in STATA version 10.0. For the IBBA data, appropriate weights were used to account for the differential recruitment of FSWs by typology within districts, differential non-response rates, and differential probabilities of selection across districts. The weighting methodology has been described previously [32], and necessary information for the calculation of sampling probabilities and design effects was recorded during the fieldwork. The primary outcome was defined as violence in the previous year. Secondary outcomes were HIV/STI prevalence, condom use (clients and regular partners) and experience of the HIV prevention program (contact with a peer educator, and contact with the drop-in centre and the project STI clinic). Odds ratios (ORs) were used as the measure of association, and the Wald chi-square test was the statistical test used. In the multivariate models, age was included as a potential confounding variable. Other potential demographic confounders were added to the model using a stepwise approach, and those which caused the ORs of the independent variables to change ≥10% were included in the final model. Multivariate analyses were performed for IBBA data only, as data from the PBS could not be linked.

## Results

### Study population and experience of violence in the past year

3,852 FSWs participated in IBBA surveys in 4 districts (1,882 FSWs at baseline and 1970 at follow-up); and 7,638 FSWs participated in 691 polling booth surveys (PBS) in 13 districts (2006: 1,659 FSWs, 176 PBS; 2007: 2,865 FSWs, 255 PBS; 2008: 3,114 FSWs, 260 PBS). Response rates for the IBBAs and PBS were high (averaging 90%).

**Figure 1:**
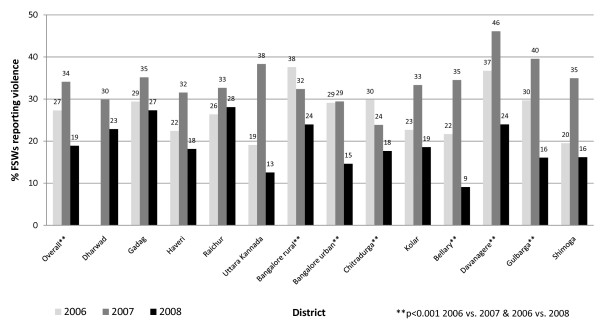
**Proportion of FSWs who report violence in the previous year, polling booth surveys 2006-2008**.

Overall, reported violence was relatively common, with 11.0% of FSWs in the IBBAs (Table 1) and 26.4% of FSWs in the PBS reporting having been beaten or raped in the past year (Figure 1). Rates of reported violence varied by district, but were generally much higher in the PBS than in the IBBA's face-to-face interviews. Participants in the IBBAs reported the main perpetrators of violence as clients (56.2%), regular partners (22.8%), "rowdies" (6.9%), the police (6.6%) and pimps (3.0%).

**Table 1:** Sociodemographic and sex work characteristics of FSWs and experience of violence in the past year^1^

	Characteristic	Not Beaten or raped past year % (n = 3439)	Beaten or raped past year % (n = 413)	P value (Wald- test)
**Current age (years)**	<25	17.8	22.5	
	25-29	22.1	26.1	
	30-34	18.7	18.9	0.04
	35-39	21.8	19.0	
	40+	19.7	13.6	
	Mean	31.9	30.3	

**Literacy**	Illiterate	65.5	64.9	0.8

**Marital status**	Never married	12.6	11.2	
	Cohabiting	1.9	5.6	
	Currently married	39.3	38.2	0.002
	Widowed/Divorced/separated/deserted	38.5	39.9	
	Devadasi	7.7	5.2	

**Additional income**	Yes	64.5	65.4	0.8

**Residency**	Local dweller	87.3	80.7	0.0007

**Sex work outside district past 6 months**	No	88.3	73.7	<0.001

	Yes	11.7	26.3	

**Sex work in Mumbai: Ever**	No	97.5	94.0	0.0002

	Yes	2.6	6.0	

**Age at first sex (years)**	<15	37.6	43.3	
	15-19	52.3	47.8	
	20-24	8.8	8.2	0.13
	25+	1.3	0.7	
	Mean	15.6	15.3	

**Age started sex work (years)**	<20	25.3	31.5	
	20-24	25.5	31.1	0.0006
	25-29	23.4	20.2	
	30+	25.8	17.2	
	Mean	24.8	23.0	

**Duration sex work (years)**	0-1	20.3	15.6	
	2 to 4	29.4	30.1	0.07
	4 to 9	22.1	27.4	
	10+	28.3	26.9	

**Usual place of solicitation**	Home	40.4	40.7	
	Brothel/lodge/dhaba	12.5	10.1	0.5
	Public places	47.1	49.2	

**Usual place of entertaining clients**	Home	61.0	52.3	
	Brothel/lodge/dabha	28.4	31.1	0.006
	Public places	10.6	16.6	

**Charge for sex (Rupees)**	< = 100	49.1	55.4	0.04
	>100	50.9	44.6	
	Mean	219	194	

**Commercial clients per week**	1 to 4	22.4	15.6	
	5 to 9	28.4	27.2	0.008
	10+	49.2	57.2	
	Mean	11.7	14.2	

**Earnings per week (from sex work)**	<500	24.5	24.7	
	501-1500	33.4	29.7	0.4
	1501+	42.2	45.6	

**Regular partner**	Regular partner	60.4	69.6	0.001

^1^Data from the integrated behavioural and biological assessments

### Experience of violence in the past year and socio-demographic associations

Socio-demographic data were collected for FSWs participating in the IBBAs only (Table 1). The mean age of respondents was 31.7 years and the majority (65.3%) were illiterate. Most were either currently married (39.2%), or were widowed, divorced, deserted or separated (38.7%). Participants had been selling sex for a mean of 7.1 years, entertained a mean of 12 clients per week (range 1-150) and charged a mean of 216 rupees ($4.50 USD) per sex act (range 10-2000 rupees). They were mainly home- (40.4%) or street- based (47.4%) FSWs, with a minority brothel-based (12.3%).

Participants who reported being beaten or raped in the past year were likely to be younger, to be either cohabiting or widowed/divorced/separated/deserted, and to have a regular partner (Table 1). They were more likely to be non-local to the area, to have sold sex outside the district in the previous six months (i.e. to be a migrant sex worker), and to have previously sold sex in Mumbai. They were also more likely to have started selling sex at a younger age (<25 years), and to entertain a greater number of clients per week (10+), compared with FSWs who did not report violence in the previous year (Table 1). FSWs who entertained clients at home were less likely to report violence than those who entertained clients in public places or in brothels/lodges/dabhas.

### Reported violence and HIV program exposure, condom use and HIV/STI rates

From the IBBA data, reported violence in the past year was associated with reduced exposure to the HIV prevention program: FSWs who reported violence were less likely to have been contacted by a peer educator or to have visited the project sexual health clinic (Table 2). In addition, they were more likely to report condom breakage in the past month compared with FSWs who did not report violence.

**Table 2:** FSW experience of violence in past year by exposure to HIV prevention program components^1^

	Not Beaten or raped past year % (n = 3439)	Beaten or raped past year % (n = 413)	Crude OR (95% CI)	P value (Wald test)	**Adjusted OR**^**2**^ (95% CI)	P value (Wald test)
Ever visited by a peer educator	89.6	84.9	0.65 (0.43, 0.97)	0.04	0.65 (0.44, 0.97)	0.04

Ever visited the drop-in centre	65.8	66.9	1.05 (0.79, 1.39)	0.7	1.08 (0.80, 1.44)	0.6

Ever visited the project sexual health clinic	68.1	59.0	0.67 (0.53, 0.86)	0.002	0.73 (0.56, 0.96)	0.02

Ever received "grey pack"^3^	53.0	50.2	0.89 (0.70, 1.14)	0.4	0.92 (0.72, 1.18)	0.5

Ever witnessed a condom demonstration	62.9	54.7	0.71 (0.55, 0.93)	0.01	0.81 (0.60, 1.09)	0.2

Condom breakage in past month	11.3	21.9	2.20 (1.69, 2.86)	<0.001	1.93 (1.46, 2.57)	<0.001

^1^Data from the integrated behavioural and biological assessments

^2^Models adjusted for survey round, age, marital status, residency, migrant, duration sex work, place entertain clients, clients per week, and regular partner.

^3^The "grey pack" comprises one gram of azithromycin and 400 mg of cefixime, and is used for the presumptive treatment of chlamydia and gonorrhea.

Violence in the past year was also strongly associated with reduced condom use with clients (but not with regular partners) (Table 3). Thus, FSWs who reported violence were less likely to report zero unprotected sex acts in the previous month, and less likely to report using a condom at last sex with both their occasional and repeat clients. As has been previously reported, condom use with regular partners was generally much less than with clients [25]. Moreover, FSWs who reported violence were significantly more likely to report anal sex, although there was no evidence to suggest this was more likely to be unprotected compared with FSWs who did not report violence (Table 3). As shown in Table 3, violence in the past year was not significantly associated with HIV infection, chlamydia or syphilis infection, but FSWs who reported being beaten or raped in the past year were more likely to be infected with gonorrhea compared with FSWs who did not.

**Table 3:** FSW experience of violence in the past year, HIV/STI rates and condom use^1^

	Not Beaten or raped past year % (n = 3439)	Beaten or raped past year % (n = 413)	Crude OR (95% CI)	P value (Wald test)	**Adjusted OR**^**2**^ (95% CI)	P value (Wald test)
HIV-1 infection	16.3	17.6	1.10 (0.80, 1.49)	0.6	0.96 (0.70, 1.32)	0.8

Syphilis	7.6	6.7	0.87 (0.57, 1.34)	0.5	0.92 (0.58, 1.46)	0.7

High-titre syphilis	3.8	3.4	0.91 (0.52, 1.59)	0.7	0.93 (0.53, 1.64)	0.8

Chlamydia infection	6.0	5.3	0.87 (0.53, 1.43)	0.6	0.93 (0.55, 1.57)	0.8

Gonorrhea infection	2.6	5.0	1.93 (1.19, 3.16)	0.008	1.93 (1.13, 3.30)	0.02

Zero unprotected sex acts in past month with clients	75.5	55.4	0.40 (0.31, 0.52)	<0.001	0.42 (0.32, 0.54)	<0.001

Condom use last sex act occasional clients	87.5	84.0	0.75 (0.53, 1.07)	0.1	0.58 (0.40, 0.85)	0.005

Condom use last sex act repeat clients^3^	83.9	71.6	0.48 (0.35, 0.67)	<0.001	0.49 (0.35, 0.70)	<0.001

Condom use last sex act regular partner^4^	37.1	40.2	1.14 (0.81, 1.61)	0.5	0.86 (0.54, 1.37)	0.5

Ever had anal sex	10.4	32.4	4.11 (3.03, 5.57)	<0.001	3.70 (2.67, 5.12)	<0.001

Condom use last anal sex^5^	63.6	54.7	0.69 (0.40, 1.19)	0.2	0.69 (0.40, 1.21)	0.2

^1^Data from the integrated behavioural and biological assessments.

^2^Models adjusted for survey round, age, marital status, residency, migrant, duration sex work, place entertain clients, clients per week and regular partner.

^3^n = 2519 ^4^n = 1555 ^5^n = 465.

### Changes over time in reported violence

From the IBBA data, at follow-up compared to baseline, there was a striking reduction in the proportion of FSWs reporting violence in the past year at the follow-up time-point (Table 4). Reductions in reported violence were seen in all districts except for Belgaum, and these reductions were significantly associated with the follow-up IBBA round in the two districts (Bellary, Shimoga) where the baseline levels of violence were highest (Table 4). Furthermore, when we categorised participants according to the length of time they reported being exposed to the HIV prevention program, FSWs who had been exposed for <12 months were more likely to report violence in the previous 12 months compared with FSWs who had been exposed to the program for one year or more (12.1% with <12 months program exposure vs. 9.2% with >12 months of exposure, AOR 0.8, 95% CI 0.6 to 1.0, p = 0.08).

**Table 4:** FSW experience of violence in the past year at baseline and follow-up surveys^1^

	Beaten or raped past year					
					
	Baseline % (n)	Follow-up % (n)	Crude OR (95% CI)	P value (Wald test)	**Adjusted OR**^**2**^ (95% CI)	P value (Wald test)
Overall	13.0 (1882)	9.0 (1970)	0.66 (0.51, 0.85)	0.001	0.70 (0.53, 0.93)	0.01

Belgaum	8.2 (386)	10.9 (402)	1.37 (0.78, 2.39)	0.2	1.31 (0.71, 2.43)	0.4

Bellary	16.7 (426)	9.6 (410)	0.53 (0.31, 0.91)	0.02	0.45 (0.27, 0.75)	0.002

Shimoga	17.9 (394)	8.6 (406)	0.43 (0.27, 0.70)	0.001	0.36 (0.19, 0.69)	0.002

Bangalore Urban	9.5 (676)	6.9 (752)	0.70 (0.44, 1.11)	0.1	0.73 (0.47, 1.15)	0.2

^1^Data from the integrated behavioural and biological assessments.

^2^Models adjusted for survey round, age, marital status, residency, migrant, duration sex work, place entertain clients, clients per week and regular partner.

When we examined changes in reported violence over time using the PBS data (Figure 1), compared with 2006, the proportion of FSWs reporting violence increased from 27.3% to 34.1% in 2007 but then fell in 2008 to 18.9%; this was significantly lower (p < 0.001) than the preceding two years (2006 vs. 2008 OR 0.62, 95% CI 0.54 to 0.71; 2007 vs. 2008 OR 0.45, 95% CI 0.40 to 0.50). Due to the unlinked anonymous methodology of the PBS, it was not possible to examine associations between violence and other variables, or to adjust for confounding. However, when we stratified the PBS data by district, by 2008, reductions in reported violence were seen across all 13 districts except one, compared with the previous two years (Figure 1).

## Discussion

We found strong evidence of a reduction in the proportion of FSWs who reported violence at follow-up compared with baseline, following the implementation of a multi-layered violence intervention as part of a wider HIV prevention program. In addition, we found that FSWs who reported experiencing violence in the past year were less likely to report using condoms with their clients, were more likely to be infected with gonorrhea, and were less likely to have accessed the HIV prevention services than FSWs who did not report violence.

There is growing recognition of the impact of physical and sexual violence on the mental and physical health of women, including HIV infection [3,13,33], and studies with non-sex workers have reported reductions in violence within intimate partnerships as part of broader HIV intervention programs [34-36]. However, although several studies have reported violence against marginalised groups such as sex workers [10,11,13,37,38], to our knowledge, this is the first study to report on a multi-layered strategy to address violence against sex workers, as part of HIV prevention programming. Consistent with studies from elsewhere, data from our IBBA surveys suggest that FSWs who reported being beaten or raped in the past year were more likely to be infected with gonorrhea than FSWs who did not [22]. The possible mechanisms through which violence can enhance vulnerability to STIs are multiple and complex, and are likely to be both direct (for example, having unprotected forced intercourse with more risky partners, or being in unsafe situations for negotiating condom use) [10,11,13,15,38]; and indirect (for example depression resulting from victimisation may reduce motivation to access HIV intervention services or negotiate condom use) [2]. Indeed, similar to findings from elsewhere, FSWs in our study who reported violence in the previous year were less likely to have been contacted by a peer educator or to have accessed the project sexual health clinics, and were much less likely to report using condoms with their clients [10]. In addition to increased knowledge and awareness of sexual risk leading to increase condom use following participation in an HIV prevention program, the conversion of a hostile environment to a more facilitative enabling environment for sex work has been reported elsewhere as a contributing factor to improved condom use [39]. Furthermore, reducing violence can translate into sex workers accessing services more easily, as well as carrying condoms without fear of their being used as evidence of sex work. In addition, working in an environment less threatened by violence supports FSWs to make choices based on their own volition [20].

As this study was evaluating a violence intervention program which has been implemented throughout Karnataka state, it was not possible for us to include a control group for comparison. With unlinked cross-sectional survey data such as these, and without a control group, it is always possible that the findings presented here represent natural trends over time and are independent of the violence intervention efforts. However, the strength and consistency of the associations across virtually all districts surveyed suggests that this is unlikely. Moreover, when we stratified participants in the IBBA surveys according to length of exposure to the HIV intervention program, violence in the preceding year was negatively associated with increasing duration of program exposure. Another limitation of the study is that it was not possible to separate out the impact on violence of individual aspects of the program, such as the policy level advocacy vs. the crisis response program vs. the police officer training vs. increasing access to social entitlements. Future qualitative and policy-oriented research would be important to undertake in the future to address this issue. Another important issue to address is program cost. It has recently been reported that the median cost per female sex worker registered in the first two years of Avahan programs in four south India states (including Karnataka) was $53, which is comparable to that of similar programs elsewhere [40]. Future work should examine the proportion of costs attributable to the components of the program directed at violence reduction.

Rates of reported violence were very high, and were much higher in the PBS compared with the IBBA, likely reflecting social desirability bias in the face-to-face interviews, and highlighting the importance of using alternative survey methods when collecting information on sensitive behaviours [6,29-31]. The elevated rates of reported violence in the 2007 PBS survey compared with the 2006 PBS survey may have reflected increased violence during this time period, as perpetrators tried to re-assert their power [20]. Alternatively, it may be that women became more comfortable with reporting violence, and more understanding of the nature of violence, and so were more likely to report it. In any case, reported violence in 2008 in the PBS was significantly lower than either of the preceding years across 12 of the 13 districts surveyed. Discussions with FSWs suggest that violence may have been under-reported [2], particularly in the baseline surveys, not least because at this time many sex workers did not consider sexual or physical violence by their intimate partners as being untoward events. Furthermore, the definition of violence used in this study was quite narrow, and thus may not have captured all violence, leading to further under-reporting. Baseline surveys were conducted 8-16 months after program initiation, by which time levels of violence may have already started to reduce, and thus the reductions in violence may have been underestimated; unfortunately, it is difficult to undertake cross-sectional surveys such as these among sex worker communities until prevention programs are underway and trust with the community is well established.

Embedding a violence intervention program such as this one within a broader HIV prevention framework has several challenges. Dealing with violence directly may conflict with power structures which are responsible for violence, and can perhaps, in the short term, put FSWs at increased risk of harm. The relationships among violence, FSWs and the perpetrators of violence are complex, with perpetrators (such as police, lovers and pimps) having the potential to act as protectors, as well as purveyors of violence [17,20]. The routine transfer of police personnel and the highly mobile nature of FSW populations suggest that in order for reductions in violence to be sustained, training of police personnel and legal empowerment training need to be ongoing.

## Conclusions

The data presented here highlight the potential for violence to undermine HIV prevention efforts, and underline the importance of addressing violence as part of wider HIV prevention programming. Such an approach can reduce the levels of violence faced by this vulnerable population, and is key to protecting the basic right of sex workers to live and work in an environment free from physical and sexual violence and abuse.

## Competing interests

The authors declare that they have no competing interests.

## Authors' contributions

TSHB performed the analyses and wrote the first draft of the paper. VG, PB, JA and HLM designed and oversaw local implementation of the intervention program. BMR and SI designed and supervised the IBBA and PBS surveys. AR, TW, JB and JFB were involved in the study design, and contributed to writing the paper. SM designed the study and contributed to writing the paper. All authors have read and approved the final manuscript.

## Pre-publication history

The pre-publication history for this paper can be accessed here:

http://www.biomedcentral.com/1471-2458/10/476/prepub
